# Immune checkpoint inhibitor induces cardiac injury through polarizing macrophages via modulating microRNA-34a/Kruppel-like factor 4 signaling

**DOI:** 10.1038/s41419-020-02778-2

**Published:** 2020-07-24

**Authors:** Wenzheng Xia, Changlin Zou, Hanbin Chen, Congying Xie, Meng Hou

**Affiliations:** 1 Department of Neurosurgery, First Affiliated Hospital, Wenzhou Medical University, Wenzhou, China; 2 Department of Neurosurgery, Xinhua Hospital Affiliated to Shanghai Jiaotong University School of Medicine, Shanghai, China; 3 Department of Radiation Oncology, First Affiliated Hospital, Wenzhou Medical University, Wenzhou, China

**Keywords:** Immunology, Cardiomyopathies

## Abstract

Cancer immunotherapy has become a well-established treatment option for some cancers; however, its use is hampered by its cardiovascular adverse effects. Immune checkpoint inhibitors (ICIs)-related cardiac toxicity took place in kinds of different forms, such as myocarditis, acute coronary syndrome, and pericardial disease, with high mortality rates. This study aimed to investigate the roles of programmed death-1 (PD-1) inhibitor, one of widespread used ICIs, in the development of murine cardiac injury. PD-1 inhibitor is known to transduce immunoregulatory signals that modulate macrophages polarization to attack tumor cells. Hence, this study explored whether the cardiovascular adverse effects of PD-1 inhibitor were related to macrophage polarization. MicroRNA-34a (miR-34a), which appears to regulate the polarization of cultured macrophages to induce inflammation, is examined in cardiac injury and macrophage polarization induced by the PD-1 inhibitor. As a target of miR-34a, Krüppel-like factor 4 (KLF4) acted as an anti-inflammation effector to take cardiac protective effect. Further, it investigated whether modulating the miR-34a/KLF4-signaling pathway could influence macrophage polarization. The PD-1 inhibitor markedly induced M1 phenotype macrophage polarization with impaired cardiac function, whereas miR-34a inhibitor transfection treatment reversed M1 polarization and cardiac injury in vivo. In vitro, PD-1 inhibitor-induced M1 polarization was accompanied by an increase in the expression of miR-34a but a decrease in the expression of KLF4. TargetScan and luciferase assay showed that miR-34a targeted the KLF4 3′-untranslated region. Either miR-34a inhibition or KLF4 overexpression could abolish M1 polarization induced by the PD-1 inhibitor. The findings strongly suggested that the PD-1 inhibitor exerted its effect in promoting M1 polarization and cardiac injury by modulating the miR-34a/KLF4-signaling pathway and inducing myocardial inflammation. These findings might help us to understand the pathogenesis of cardiac injury during immunotherapy, and provide new targets in ameliorating cardiac injury in patients with cancer receiving PD-1 inhibitor treatment.

## Introduction

Immune checkpoint inhibitors (ICIs), including anti-CTLA-4 and anti-programmed death-1 (PD-1), can induce tumor responses in both solid and hematological malignancies^1,2^. Many of these tumor responses are durable over the years^3^. Although immune checkpoint-modulating antibodies have revolutionized clinical immunotherapy, their application is restrained by a spectrum of immune-related adverse events^4,5^. A large number of systems have been confirmed to be potential targets of immune-related toxicity induced by immune checkpoint-blocking antibodies^6^, including colitis, hepatitis, rash, and endocrinopathies^7^. Cardiac injury has been implicated as a severe outcome in a subset of patients^8^. Therefore, a more detailed understanding of immune-related cardiotoxicity associated with immune checkpoint-blocking antibodies may be helpful when patients with cardiac injury receive immune therapy.

Treatment with ICIs not only enhances immune reactions to tumor cells but is also frequently associated with inflammatory reactions to nontumor cells^9^. Pathologically, immune-related adverse events in the setting of ICIs treatment are largely characterized by the infiltration of macrophages into the tissue, causing injury^10^. Recent data suggest the involvement of macrophage polarization in cardiovascular diseases, such as chemotherapy-related cardiac injury^11^. Under some condition, monocytes travel to the site of injury and undergo differentiation into either proinflammatory M1 macrophages or antiinflammatory M2 macrophages^12^. In the chemotherapy-related cardiotoxicity, modulating macrophage polarization could partially alleviate cardiac injury^13^. However, whether ICIs-related cardiotoxicity involves macrophage polarization is unknown.

MicroRNAs (miRs) are endogenous, small, noncoding RNAs that modulate gene expression post-transcriptionally by binding to the 3′-untranslated region (3′-UTR) of target genes^14^. MiRs play important roles in the pathogenesis of various heart diseases, including coronary heart diseases, heart failure, and immune myocarditis^15,16^. In particular, numerous miRNAs take part in cardiotoxicity by modulating macrophage polarization^17^. Therefore, miRNAs hold great promise as diagnostic biomarkers and therapeutic targets for ICIs-related cardiac complications.

MiR-34a is abundant in mammalian cardiac tissues, especially senescent heart tissue^18^. It regulates cardiac ischemia injury by modulating macrophages^19^. Generally, it exerts cardiotoxicity by modulating immune reaction^20^. However, whether this cardiac damage-related miR also has an adverse effect in the ICIs-related cardiotoxicity remains elusive.

Krüppel-like factor 4 (KLF4), a DNA-binding transcriptional regulator, which is highly expressed in monocytes, modulates a wide range of biologic activities^21^. Noticeably, it has an important role in cardiac protection, such as inhibiting KLF4 leading to cardiac hypertrophy^22^. Smooth muscle cell-specific KLF4 knockout at baseline resulted in cardiac dilation^23^. Moreover, KLF4-deficient macrophages demonstrated increased proinflammatory gene expression, indicating that KLF4 is essential for polarization^24^. Interestingly, KLF4 is an essential regulator of macrophage polarization in heart tissues, suppressing M1 polarization and thus relieving cardiac inflammation and atherosclerosis^25^. However, whether the interplay occurs between ICIs-related cardiac injury and KLF4 remains unknown. Therefore, it was presumed that KLF4 might take part in mitigating ICIs-induced cardiotoxicity, as a target of miR-34a.

The purpose of this study was to evaluate whether PD-1 inhibitor, as a common ICIs, could induce cardiotoxicity by eliciting proinflammatory M1 macrophages. It also examined whether this process was mediated through the miR-34a/KLF4 pathway. Furthermore, it aimed to understand whether modulating miR-34a could improve cardiac function after PD-1 treatment.

## Materials and methods

### Animals

Male C57/Bl6 mice were maintained in accordance with guidelines published by the US National Institutes of Health. All study procedures were approved by the Institutional Animal Care and Use Committee of Wenzhou Medical University. This study was conducted in compliance with the Guide for the Care and Use of Laboratory Animals published by the National Academy Press (NIH, revised in 1996).

### Treatment of PD-1 inhibitor in vivo

Male eight-week-old C57/Bl6 mice were treated with an In Vivo Plus anti-mouse PD-1 inhibitor (Bioxcell, West Lebanon, USA), intraperitoneally, at a concentration of 5 mg/kg, on days 1 and 14 of the treatment cycle (28 days a cycle). We finished the intraperitoneal injection in 15 s.

### Echocardiographic evaluation

Twenty-eight days after first treatment, mice inhaled anesthetic isoflurane and vevo 2100 was applied to observe the echocardiographic parameters of mice to assess the cardiac functions (Vevo TM 2100, Visual Sonics, Canada). Short axis was used to measure left ventricular internal diameter; diastole (LVID;d), left ventricular internal diameter; systole (LVID;s), left ventricular end-diastolic volume (LVEDV), left ventricular end-systolic volume (LVESV). The values of ejection fraction (EF) and fractional shortening (FS) were calculated according to the following equation: EF% = [(LVEDV − LVESV)/LVEDV] × 100%; FS% = [(LVID;d − LVID;s)/LVID;d] × 100%.

### Immunofluorescence staining

Deparaffinized tissue sections were stained with antibodies for Sarcomeric-α-actin (1:50, Abcam, ab9465). Thereafter, the inducible nitric oxide synthase (iNOS) were used to detect M1 macrophages (1:50; Abcam, ab15323). Following primary incubation, slides were washed with 1× phosphate-buffered saline and incubated with Alexa Fluor 594 (1:50; Invitrogen, A32744) or Alexa Fluor 488 (1:50; Invitrogen, A32731) secondary for 1 hour at room temperature. Sections were counterstained with 4′,6-diamidino-2-phenylindole mounting medium and analyzed. Fluorescence was detected under a microscope.

### Quantitative reverse transcription-polymerase chain reaction (qRT-PCR)

QRT-PCR was performed as described previously^26^. The primers are listed in Table 1. In brief, total tissue and cellular RNA was extracted by TRIzol reagent and reverse transcribed to cDNA and then amplified using the SYBR-Green master mix kit. All procedures were performed in triplicate. The mRNA levels were calculated relative to the control Gapdh or U6 using the 2^−ΔΔCq^ method.

**Table 1:** Primer sequences.

Genes	Sequences
iNOS	F: 5′-GCATCCCAAGTACGAGTGGT-3′
	R: 5′-CCATGATGGTCACATTCTGC-3′
IL-1β	F: 5′-GGAGAAGCTGTGGCAGCTA -3’
	R: 5′-GCTGATGTACCAGTTGGGGA-3′
IL-6	F: 5′-CCGGAGAGGAGACTTCACAG -3’
	R: 5′-TGGTCTTGGTCCTTAGCCAC -3’
TNF-α	F: 5′-GACCCTCACACTCAGATCAT-3′
	R: 5′-TTGAAGAGAACCTGGGAGTA-3′
Arg1	F: 5′-AGCACTGAGGAAAGCTGGTC -3’
	R: 5′-CAGACCGTGGGTTCTTCACA -3’
TGF-β1	F: 5′-GCGTATCAGTGGGGGTCA -3’
	R: 5′-GTCAGACATTCGGGAAGCAG-3′
IL-10	F: 5′-ACAGCCGGGAAGACAATAAC-3′
	R: 5′-CAGCTGGTCCTTTGTTTGAAAG-3′
miR-34a	F: 5′-GGCCAGCTGTGAGTGTTTCTTTGG-3′
	R: 5′-CTCGCTTCATCTTCCCTCTTGGG-3′
KLF4	F: 5′-CTGAACAGCAGGGACTGT-3′
	R: 5′-GTGTGGGTGGCTGTTCTTTT-3′
GAPDH	F: 5′-GGA GCC AAA AGG GTC ATC AT-3′
	R: 5′-GTG ATG GCA TGG ACT GTG GT-3′
U6	F: 5′-CTCGCTTCGGCAGCACA-3′
	R: 5′-AACGCTTCACGAATTTGCGT-3′

### Microarray analysis

Heart tissue from mice treated with or without PD-1 inhibitor were lysed immediately in 500 μL TRIzol (ThermoFisher Scientific, Waltham, MA, USA) and stored at −80 °C before purification using a standard phenol–chloroform extraction protocol with an RNAqueous Micro Kit (ThermoFisher Scientific). The transcriptome was subjected to microarray analysis using an Affymetrix human array (ThermoFisher Scientific) and normalized based upon quantiles.

### Antagomir studies

Chemically modified antisense oligonucleotides designed to target miR-34a. Eight-week-old male C57/Bl6 mice were subjected to sham or PD-1 inhibitor treatment. After the same, mice were injected (intraperitoneally) with antagomir-34a (80 mg/kg) or control antagomir (80 mg/kg) for 3 consecutive days as previously reported^27^.

### Local in vivo transfection

Adeno-associated virus serotype 9 (AAV9) vectors were used for overexpression of KLF4 in vivo (JiKai Gene, Jiangsu, China). The recombinant AAV9 virus carrying KLF4 cDNA was used. In brief, 8-week-old mice were used for the AAV virus injection through jugular vein at a dose of 1 × 10^13^ vg per kg body weight, as previously reported^28^.

### Cell culture and cell treatment

HL-1 murine cardiomyocytes were a kind gift of Dr. William C. Claycomb. Cells were maintained in fibronectin-coated flasks, supplemented with 10% FBS, 100 U/mL penicillin, 100 mg/mL streptomycin, and 2 mm l-glutamine and kept semi-confluent at all times. The PD-1 inhibitor treatment was carried out by exposing the cell culture to concentration of 5 µg/ml PD-1 inhibitor for short periods of time, lasting 1 hour^29^.

### Cell proliferation assay

The rate of cell proliferation was estimated using the cell counting kit-8 (CCK-8) assay, which was performed according to the manufacturer’s protocol. In brief, cells grown in a 96-well plate were incubated with the CCK-8 solutions for 1 h at 37 °C, following which the absorbance of each well at 450 nm was recorded.

### MTT assay

The MTT assay was used to determine cell viability. In brief, 300 µL of MTT reagent was added to each well 2 h prior to harvesting. The supernatant was then removed and incubated with 400 µL of dimethyl sulfoxide for 10 min. Absorbance at 540 nm was recorded using an enzyme-linked immunosorbent assay plate reader. Three repeats were performed.

### Cell cycle assay

The cells, fixed with 70% cold anhydrous ethanol, were treated with propidium iodide (Sigma, St. Louis, MO, USA) with RNase A. Flow cytometer equipped with Cell Quest software was utilized to detect the cell cycle distribution.

### Flow cytometric analysis of cell apoptosis

Apoptosis was determined by detecting phosphatidylserine exposure on cell plasma membranes using an Annexin V-FITC Apoptosis Detection Kit, according to manufacturer’s protocol, as previously reported^30^.

### Isolation of cardiac macrophages

Cardiac macrophages were isolated as previously described^31^. In brief, hearts were excised, washed in warmed Hanks Balanced Salt Solution (HBSS), placed in warm digestion buffer containing Liberase TH (5 mg/ml, Roche 05,401,135,001) and DNase1 (2000 units, Sigma D4263) for 5 min, triturated with a pipet and passed through a 40 µm filter into stopping buffer containing 10% fetal bovine serum (FBS) in 5 ml HBSS. Cells were centrifuged, supernatant discarded and red blood cells lysed with ACK lysing buffer (Gibco, A10492-01). To isolate macrophages, cells were incubated with 50 µl of anti-CD45 (mouse) or anti-CD14 (human) magnetic beads for 30 min. Cells were then allowed to flow through the magnetic-activated cell sorting magnetic column with magnet in place.

### Flow cytometry

To analyze the macrophage polarization, harvested cells were washed with PBS, and incubated at 4 °C for 30 min in PBS/bovine serum albumin with anti-F4/80 and anti-iNOS, anti-CD206, anti-CD38, or isotype controls. Finally, cells were washed and fixed in 4% paraformaldehyde. Fluorescence was measured by flow cytometry and analyzed using FACSDiva Pro Software (Becton-Dickinson, San Jose, CA).

### Mir-34a inhibition in vitro

The macrophages were seeded into six-well plates at a density of 1 × 10^5^ cells per well and incubated for 12 h. To induce the inhibition of miR-34a, the cells were transfected with miR-34a inhibitor or negative control (NC) inhibitor (Pre-miR miRNA Precursors, Life Technologies, Karlsruhe, Germany) using X-treme transfection reagent (Roche Applied Science, Penzberg, Germany), according to the manufacturer’s protocol. The cells were harvested for further analysis 48 h after transfection, and the transfection efficiency was analyzed using qRT-PCR.

### Luciferase reporter assay

The 3′-UTRs of KLF4 were synthesized to induce the mutagenesis of KLF4. The macrophages were seeded into a 24-well plate. After overnight culture, the cells were co-transfected with equal amounts of a wild-type (WT) or mutated plasmid and miR-34a mimic or miR-NC mimic. Luciferase assays were performed using a dual-luciferase reporter assay system (Promega) 24 h after transfection.

### Western blot analysis

The cells were harvested, and total protein was extracted using radioimmunoprecipitation assay buffer solution. The protein samples were denatured, separated by 10% sodium dodecyl sulfate–polyacrylamide gel electrophoresis, and transferred to polyvinylidene difluoride membranes. The membranes were incubated with KLF4 (ab214666, 1:1000) and β-actin (ab179467, 1:1000) primary antibodies overnight at 4 °C. The membranes were further incubated with IgG-horseradish peroxidase goat anti-rabbit secondary antibody (ab7090: 1:2000) for 2 h at room temperature. The signals were developed by enhanced chemiluminescence (CST, #6883). The stained protein bands were visualized using a Bio-Rad ChemiDoc XRS imaging system and analyzed using Quantity One software.

### Transient transfection

For overexpression of KLF4 in macrophages, they were transduced with adenoviral KLF4 (Ad-KLF4) or adenoviral control (Ad-Ctrl) as described previously^32^. The transfection efficiency was confirmed by qRT-PCR and western blot.

### Statistical analysis

Data were expressed as the mean ± standard deviation (SD). Differences between groups were tested by one-way analysis of variance, and comparisons between two groups were evaluated using the Student *t* test. Analyses were performed using SPSS package v19.0 (SPSS Inc., IL, USA). A *P* value < 0.05 was considered statistically significant.

## Results

### PD-1 inhibitor impaired heart function accompanied by the inducement of differentiation of M1 macrophages

**Fig. 1:**
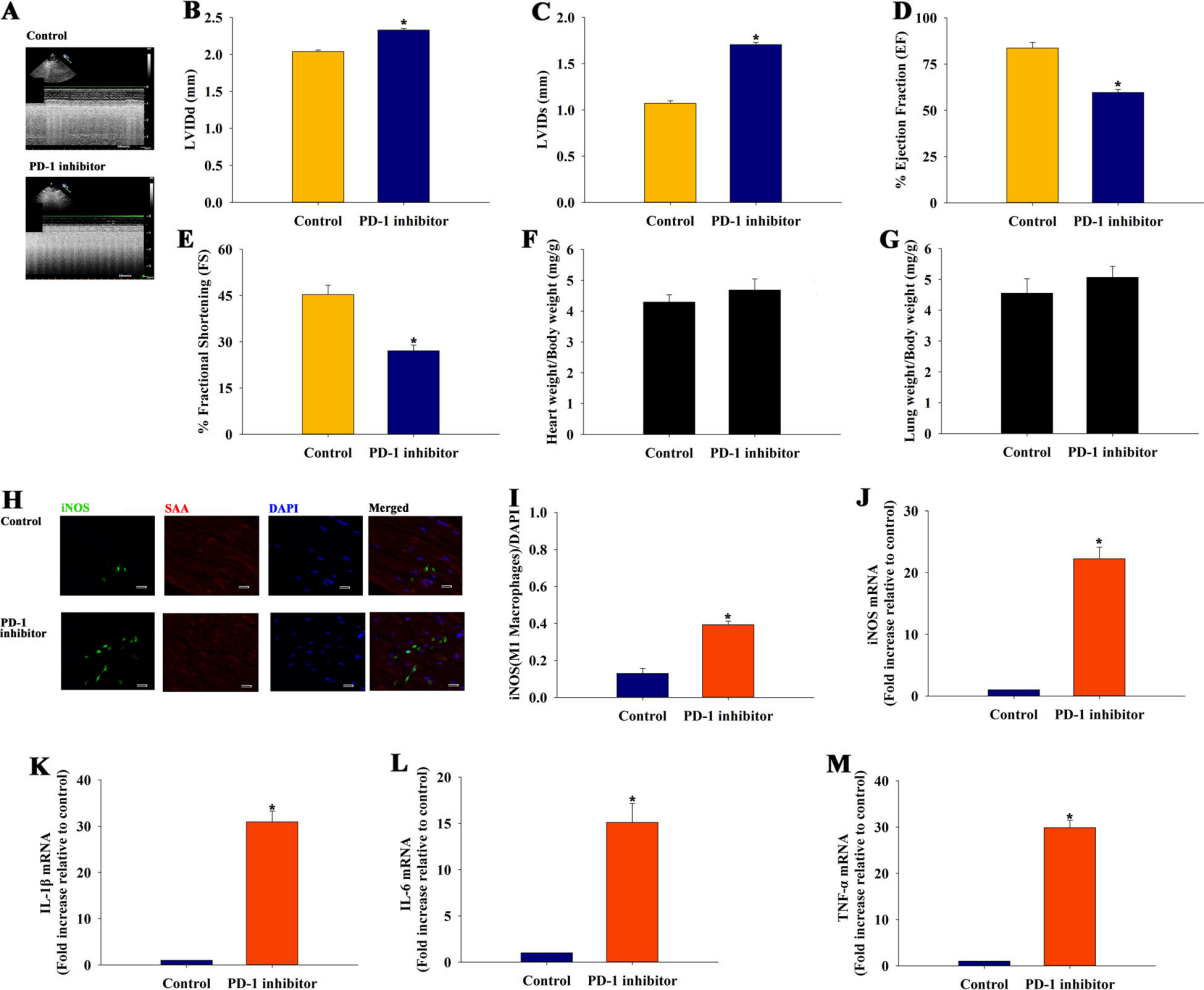
PD-1 inhibitor impaired the heart function accompanied by the inducement of differentiation of M1 macrophages. **a** Representative images of echocardiography exhibiting changes in cardiac function in each group. Echocardiographic analysis of left ventricular end-diastolic diameter (LVIDd) **b**, left ventricular end-systolic diameter (LVIDs) **c**, ejection fraction (EF) **d**, and fractional shortening (FS) **e** in week 4 after the first cycle of PD-1 inhibitor treatment or sham operation, *n* = 6 per group. **f** Ratio of heart weight to body weight. **g** Ratio of lung weight to body weight; *n* = 6 per group. **h** Representative photomicrographs of iNOS. **i** Quantitative analysis of iNOS-positive M1 proinflammatory macrophages, *n* = 3 per group; Scale bar: 50 μm. Proinflammatory cytokine iNOS **j**, IL-1β **k**, IL-6 **l**, and TNF-α **m** mRNA expression levels were examined using qRT-PCR, *n* = 6 per group. ^*^P < 0.05 versus the control group.

Whether PD-1 inhibitor impaired heart function by modulating macrophage polarization was investigated in a murine model. Echocardiography showed that left ventricular ejection fraction and FS significantly decreased in the PD-1 inhibitor group compared with the sham group (Fig. 1a–e). However, there was no difference in ratio of heart weight to body weight and lung weight to body weight (Fig. 1f, g). Immunofluorescence staining was performed for iNOS to evaluate whether PD-1 inhibitor treatment influenced M1 macrophage populations (Fig. 1h). The number of iNOS-positive cells (M1 macrophages) significantly increased in the PD-1 inhibitor-treated animals compared with the controls (Fig. 1I). M1 macrophages have been shown to be upregulated in murine hearts undergoing cardiac proinflammation. Therefore, qRT-PCR was performed to determine whether PD-1 inhibitor treatment increased the levels of proinflammatory cytokines. As shown in Fig. 1j–1m, the levels of proinflammatory cytokines iNOS (Fig. 1j), IL-1β (Fig. 1k), IL-6 (Fig. 1l), and TNF-α (Fig. 1m) were induced by the PD-1 inhibitor in the heart.

### MiR-34a took effect in cardiac injury and macrophage M1 phenotype polarization elicited by the PD-1 inhibitor in vivo

**Fig. 2:**
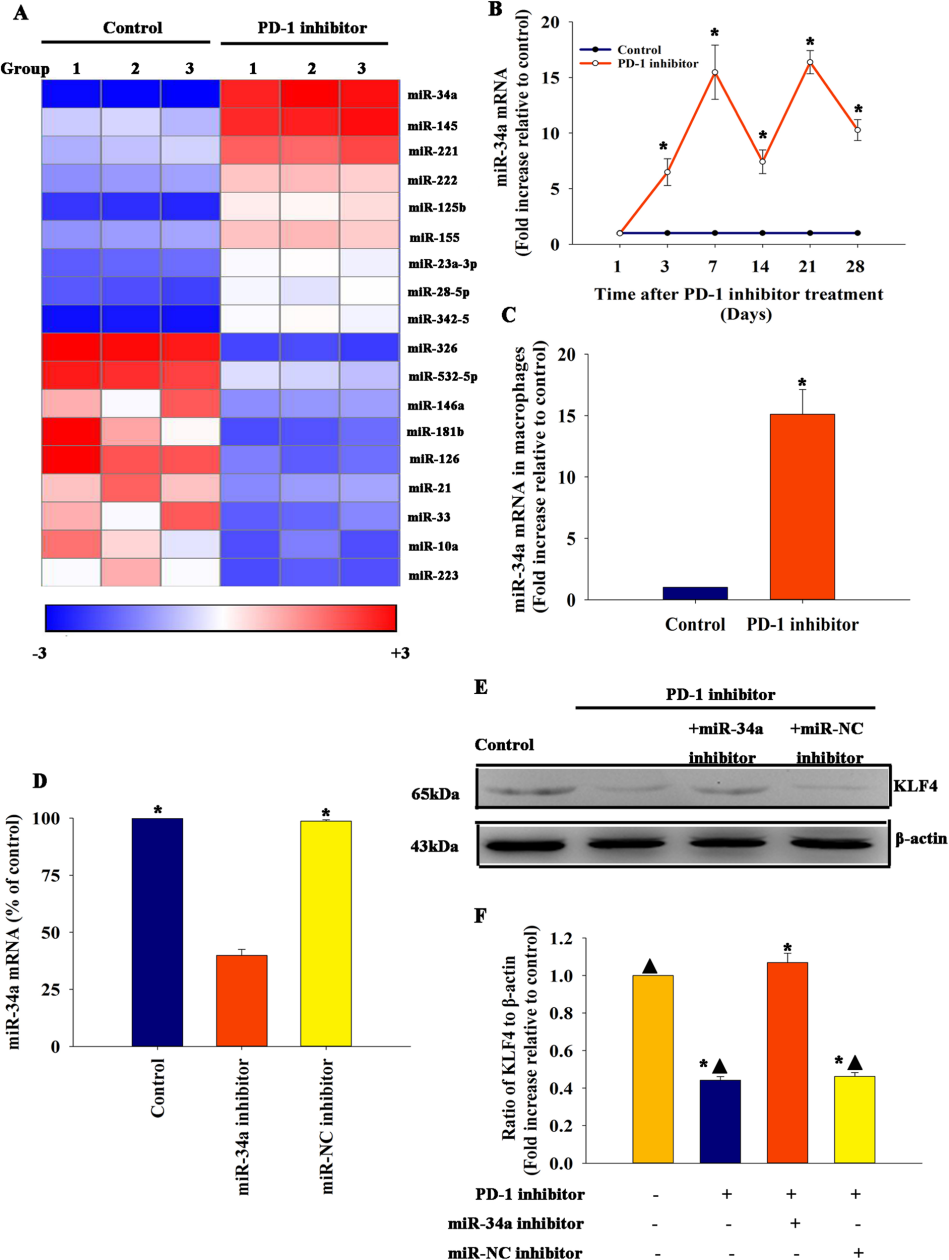
MiR-34a was elicited when mice were treated with a PD-1 inhibitor. **a** Heat map of miRs differentially expressed between mice treated with a PD-1 inhibitor and control mice, *n* = 3 per group. **b** MiR-34a expression was validated using qRT-PCR in mice treated with a PD-1 inhibitor and control mice. *n* = 6 per group ^*^P < 0.05 versus the control group. **c** MiR-34a expression was validated using qRT-PCR in macrophages isolated from mice treated with a PD-1 inhibitor and control mice. *n* = 3 per group ^*^P < 0.05 versus the control group. Mouse hearts were locally transfected with an inhibitor control (miR-NC inhibitor) or an miR-34a inhibitor. Untreated mice were used as a control. **d** Transfection efficiency was analyzed using qRT-PCR. *n* = 3 per group. ^*^P < 0.05 versus miR-34a inhibitor. **e**, **f** Expression of KLF4 from hearts locally transfected with an inhibitor control (miR-NC inhibitor) or an miR-34a inhibitor in mice treated with a PD-1 inhibitor, only PD-1 inhibitor-treated mice, or untreated mouse heart tissue was examined by western blot analysis.compared with control mice *n* = 3 per group. ^*^P < 0.05 versus the control group; ^▲^*P* < 0.05 versus the PD-1 inhibitor + miR-34a inhibitor group.

Studies suggested that miRs had an intriguing role in macrophage polarization^33^. Therefore, this study aimed to investigate whether miRs contributed to the immunomodulatory effect of PD-1 inhibitor in cardiac injury. Microarray analysis was performed between the sham group and PD-1 inhibitor group to understand how a PD-1 inhibitor influenced macrophage polarization (Fig. 2a). As shown in Fig. 2a, miR-34a was abundant in hearts treated with a PD-1 inhibitor and might have a correlation with cardiac injury and macrophage polarization. QRT-PCR further confirmed that miR-34a was more abundant in hearts treated with a PD-1 inhibitor in a time-dependent manner (Fig. 2b). At meanwhile, miR-34a expression in the macrophages isolated from mice treated with PD-1 inhibitor was increased, when compared with control (Fig. 2c). KLF4 is a critical regulator of macrophage M1/M2 polarization and also acts as an important target of miR-34a^34^. We found that the PD-1 inhibitor impaired the expression of KLF4 in the heart tissue, whereas the inhibition of miR-34a in vivo (Fig. 2d) recovered the KLF4 expression in the heart (Fig. 2e, f).

**Fig. 3:**
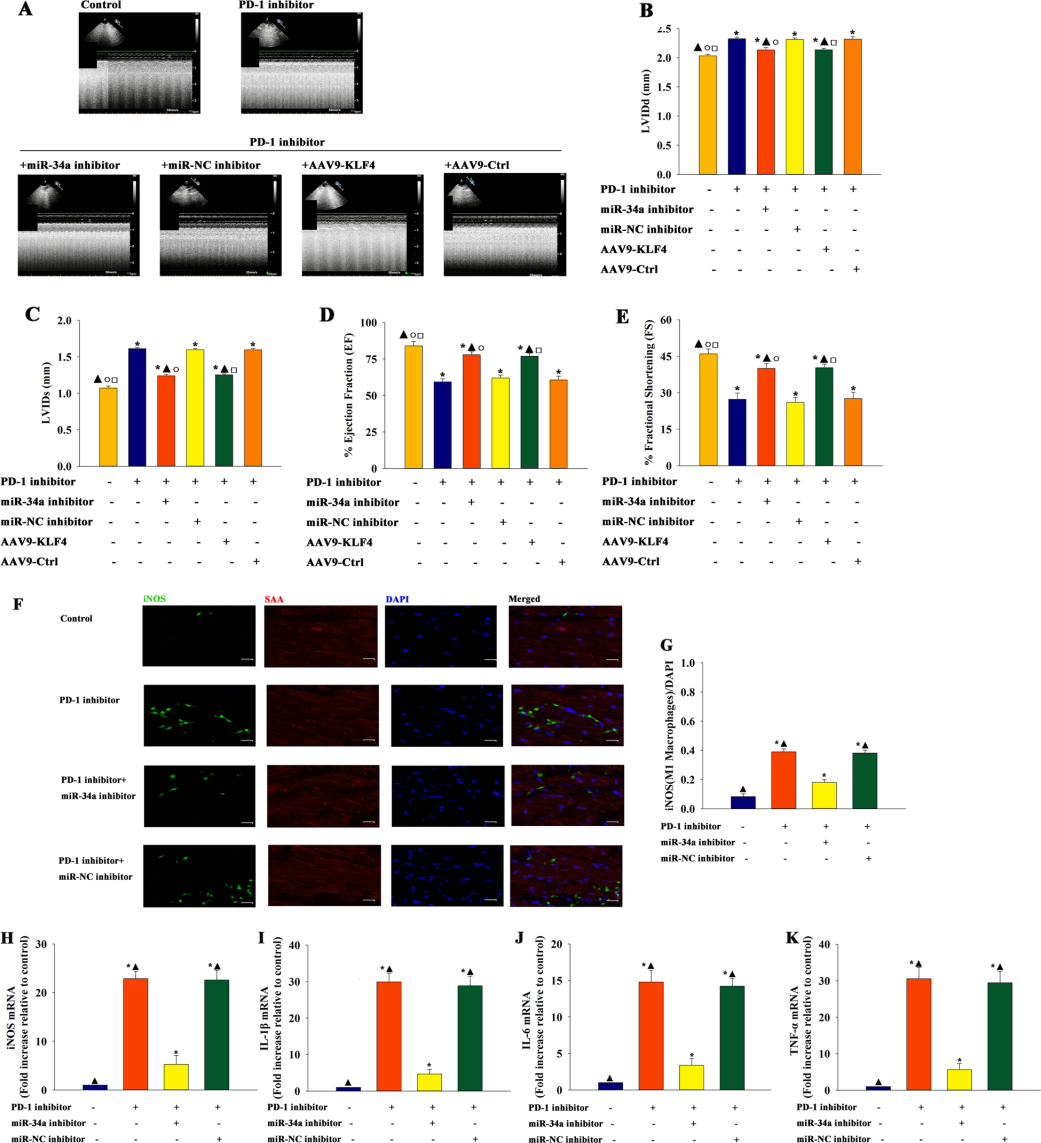
MiR-34a took effect in cardiac injury and macrophage M1 phenotype polarization elicited by a PD-1 inhibitor in vivo. Mouse hearts were locally transfected with miR-34a inhibitor, miR-NC inhibitor, AAV9-KLF4, or AAV9-Control (AAV9-Ctrl) separately, then treated with PD-1 inhibitor. The mice were treated with a PD-1 inhibitor. Untreated mice were used as control. **a** Representative images of echocardiography exhibiting changes in cardiac function in each group. Echocardiographic analysis of left ventricular end-diastolic diameter (LVIDd) **b**, left ventricular end-systolic diameter (LVIDs) **c**, ejection fraction (EF) **d**, and fractional shortening (FS) **e** in week 4 after the first cycle of PD-1 inhibitor treatment or sham operation. *n* = 3 per group. ^*^P < 0.05 versus the control group; ^▲^*P* < 0.05 versus the PD-1 inhibitor group. ○*P* < 0.05 versus the PD-1 inhibitor + miR-NC inhibitor group; ^□^*P* < 0.05 versus the PD-1 inhibitor + AAV9-Ctrl group. **f** Representative photomicrographs of iNOS. **g** Quantitative analysis of iNOS-positive M1 proinflammatory macrophages. *n* = 3 per group. Scale bar: 50 μm. Pro-inflammatory cytokine iNOS **h**, IL-1β **i**, IL-6 **j**, and TNF-α **k** mRNA expression levels were examined using qRT-PCR. *n* = 6 per group. ^*^P < 0.05 versus the control group; ^▲^*P* < 0.05 versus the PD-1 inhibitor + miR-34a inhibitor group.

Monocytes have been well documented as cells with the ability to differentiate into M1 proinflammatory or M2 antiinflammatory macrophage subpopulations^35^. MiR-34a inhibitor transfection was performed in vivo to evaluate whether miR-34a inhibition treatment influenced M1 macrophage populations and cardiac function modulation by PD-1 inhibitor. Echocardiography data revealed that PD-1 inhibitor-treated mice had significant impairment in cardiac function compared with control mice. However, mice treated with PD-1 inhibitor + miR-34a inhibitor showed recovery in cardiac function. At meanwhile, AAV9-KLF4 vectors were employed to induce KLF4 overexpression in vivo. Mice treated with PD-1 inhibitor + AAV9-KLF4 showed recovery in cardiac function (Fig. 3a–e). The number of iNOS-positive cells (M1 macrophages) significantly increased in PD-1 inhibitor-treated animals, compared with controls. Moreover, iNOS levels were significantly reduced in the hearts of animals in the PD-1 inhibitor + miR-34a inhibitor-treated group compared with the PD-1 inhibitor group, but no change is observed in the hearts of animals in the PD-1 inhibitor + miR-NC inhibitor group (Fig. 3f, g). M1 macrophages have shown to be upregulated in murine hearts undergoing cardiac proinflammation. Therefore, qRT-PCR was performed to determine whether miR-34a inhibitor treatment reduced the expression levels of proinflammatory cytokines. As shown in Fig. 3h–k, the expression levels of proinflammatory cytokine iNOS (Fig. 3h), IL-1β (Fig. 3i), IL-6 (Fig. 3j), and TNF-α (Fig. 3k) induced by the PD-1 inhibitor decreased when the expression of miR-34a was inhibited in the heart.

### PD-1 inhibitor does not cause cardiomyocyte injury

The proliferation under PD-1 treatment was examined first to investigate the potentially harmful effects of PD-1 inhibitor on cardiomyocytes. The proliferation was not hampered when HL-1 cardiomyocytes were exposed to the PD-1 inhibitor (Fig. S1A). However, the administration of the PD-1 inhibitor did not alter cellular vitality (Fig. S1B) and cell cycle (Fig. S1C, D). Also, the PD-1 inhibitor did not induce cellular apoptosis (Fig. S1E, F). These results indicated that the PD-1 inhibitor alone did not affect the biological activity of the cardiomyocytes. Interestingly, PD-1 inhibitor alone couldn’t increase miR-34a expression in the cardiomyocytes (Fig. S1G).

### PD-1 inhibitor regulated macrophage polarization to an M1 phenotype

**Fig. 4:**
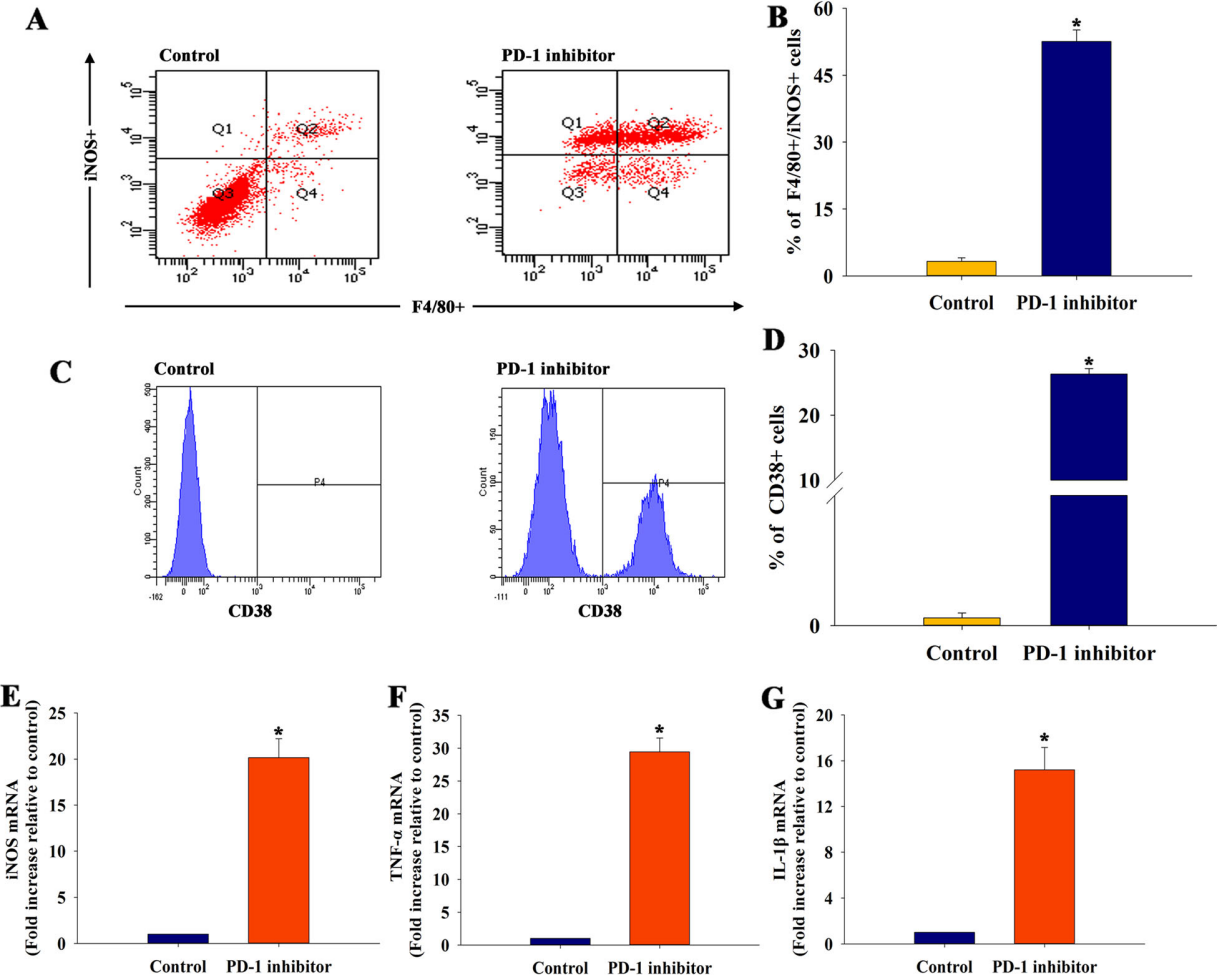
PD-1 inhibitor regulated macrophage polarization to the M1 phenotype in vitro. **a** The typical results of F4/80+/iNOS+ type using flow cytometry in macrophages treated with conditioned medium with or without a PD-1 inhibitor for 3 days. **b** Quantitative analysis of F4/80+/iNOS+ cells were analyzed using flow cytometry. **c** The typical results of CD38+ type using flow cytometry in macrophages treated with conditioned medium with or without a PD-1 inhibitor for 3 days. **d** Quantitative analysis of CD38+ cells were analyzed using flow cytometry. *n* = 3 per group. The expression levels of M1 markers iNOS **e**, TNF-ɑ **f**, and IL-1β **g** mRNAs were examined using qRT-PCR. *n* = 6 per group. ^*^P < 0.05 versus the control group.

Considering the immune-regulatory effect of PD-1 inhibitor in vivo, it was hypothesized that a PD-1 inhibitor could influence macrophage polarization in vitro. The macrophage phenotype induced by a PD-1 inhibitor was analyzed by flow cytometry. The PD-1 inhibitor-induced M1 polarization with positive F4/80+/iNOS+ (Fig. 4a, b) and CD38+ expression (Fig. 4c, d), but with negative CD206+ expression (Fig. S2A, B). The expression of M1 and M2 markers at the mRNA level was tested using qRT-PCR. The results showed a significant increase in the levels of M1 markers (iNOS, TNF-ɑ, and IL-1β) (Fig. 4e–g), but no influence of M2 markers (Arg1, TGF-β1, and IL-10) (Fig. S2C–E). Taken together, these data implied that the presence of PD-1 inhibitor regulated macrophage polarization to the M1 phenotype.

### MiR-34a was a candidate effector of PD-1 inhibitor-mediated macrophage polarization

**Fig. 5:**
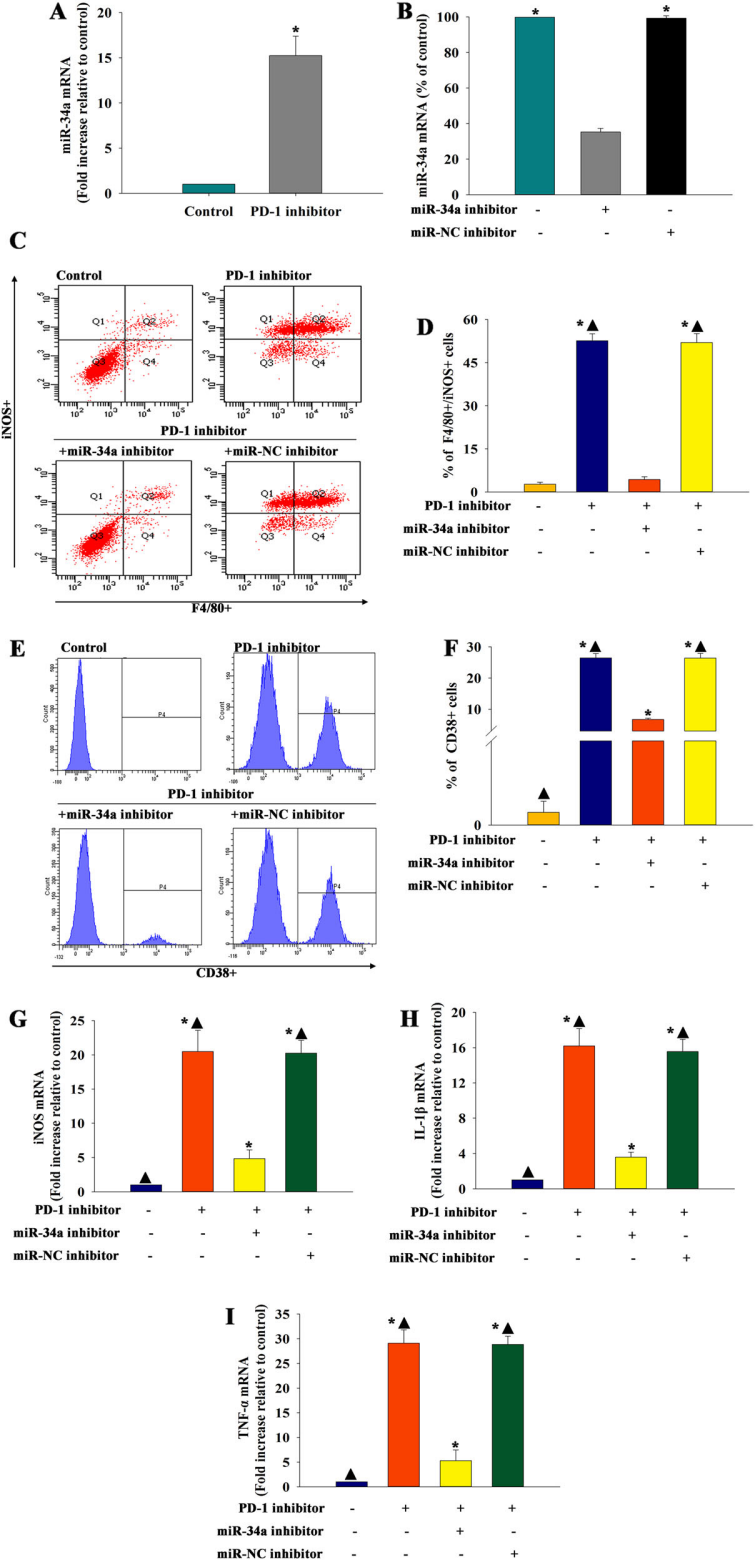
MiR-34a was a candidate effector of PD-1 inhibitor-mediated macrophage polarization. **a** qRT-PCR validation of differentially regulated miR-34a in macrophages, with or without PD-1 inhibitor treatment. *n* = 3 per group. ^*^P < 0.05 versus control. **b** Macrophages were transfected with an miR-NC inhibitor or an miR-34a inhibitor. Untreated macrophages were used as a control. Transfection efficiency was analyzed using qRT-PCR. *n* = 3 per group. ^*^P < 0.05 versus the miR-34a inhibitor group. **c** The typical results of F4/80+/iNOS+ type using flow cytometry in macrophages. **d** Quantitative analysis of F4/80+/iNOS+ cells were analyzed using flow cytometry. **e** The typical results of CD38+ type using flow cytometry. **f** Quantitative analysis of CD38+ cells were analyzed using flow cytometry. *n* = 3 per group. The expression levels of M1 markers iNOS **g**, IL-1β **h**, and TNF-α **i** mRNAs were examined using qRT-PCR. *n* = 6 per group. ^*^P < 0.05 versus the control group; ^▲^*P* < 0.05 versus the PD-1 inhibitor+miR-34a inhibitor group.

The in vivo study suggested that miR-34a had an intriguing role in cardiac injury and macrophage polarization induced by a PD-1 inhibitor. Therefore, the study investigated whether miR-34a contributed to the immunomodulatory effect of PD-1 inhibitor in vitro. QRT-PCR confirmed that miR-34a was more abundant in macrophages treated with PD-1 inhibitor (Fig. 5a).

The function of miR-34a was inhibited by transfecting macrophages with an miR-34a inhibitor to confirm the role of miR-34a in PD-1 inhibitor-induced macrophage polarization, which was subsequently confirmed by qRT-PCR. The qRT-PCR analysis demonstrated that the level of miR-34a was significantly reduced in miR-34a inhibitor-treated macrophages compared with NC inhibitor-treated macrophages (Fig. 5b). Then, PD-1 inhibitor-stimulated macrophages were treated with an NC inhibitor or an miR-34a inhibitor, and the cells were collected for flow cytometry. The results showed that the PD-1 inhibitor-induced macrophage polarization to the M1 phenotype, whereas the inhibition of miR-34a blunted the inducement; however, the miR-NC inhibitor had no effect (Fig. 5c–f). Also, the levels of M1 markers (iNOS, TNF-ɑ, and IL-1β) decreased in the PD-1 inhibitor + miR-34a inhibitor group (Fig. 5g–i) compared with the PD-1 inhibitor group.

### MiR-34a elicited by a PD-1 inhibitor modulated the macrophage phenotype through targeting KLF4

**Fig. 6:**
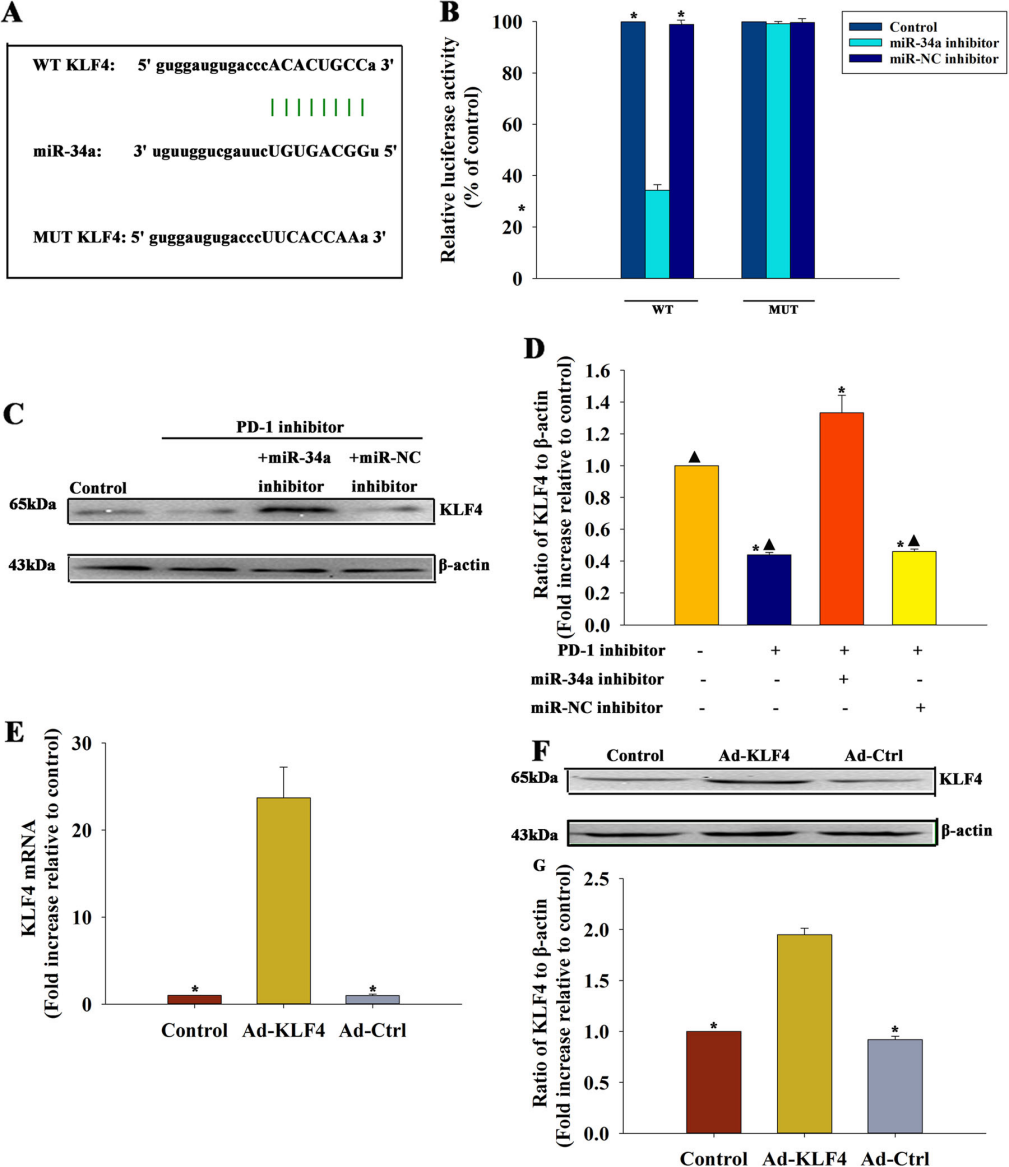
MiR-34a directly targeted KLF4. **a** Predicted binding sites between miR-34a and KLF4 3’-UTR. **b** Dual-luciferase assay was performed in macrophages after co-transfection with KLF4 3’-UTR WT or mutant (MUT) plasmids, and miR-34a mimics or miR-NC mimics. *n* = 3 per group. ^*^P < 0.05 versus miR-34a mimic in the WT group. **c**, **d** Western blot analysis of KLF4 and β-actin protein levels in macrophages transfected with an inhibitor control or an miR-34a inhibitor and treated with a PD-1 inhibitor. Macrophages were treated with a PD-1 inhibitor. Untreated macrophages were used as control. *n* = 3 per group. ^*^P < 0.05 versus the control group; ^▲^*P* < 0.05 versus the PD-1 inhibitor + miR-34a inhibitor group. **e**–**g** Macrophages were transfected with Ad-KLF4 or Ad-Ctrl as a control. Transfection efficiency was determined using qRT-PCR **e** and western blot analysis **f**, **g**. *n* = 3 per group. Each column represents the mean ± standard deviation from three independent experiments. ^*^P < 0.05 versus the Ad-KLF4 group.

**Fig. 7:**
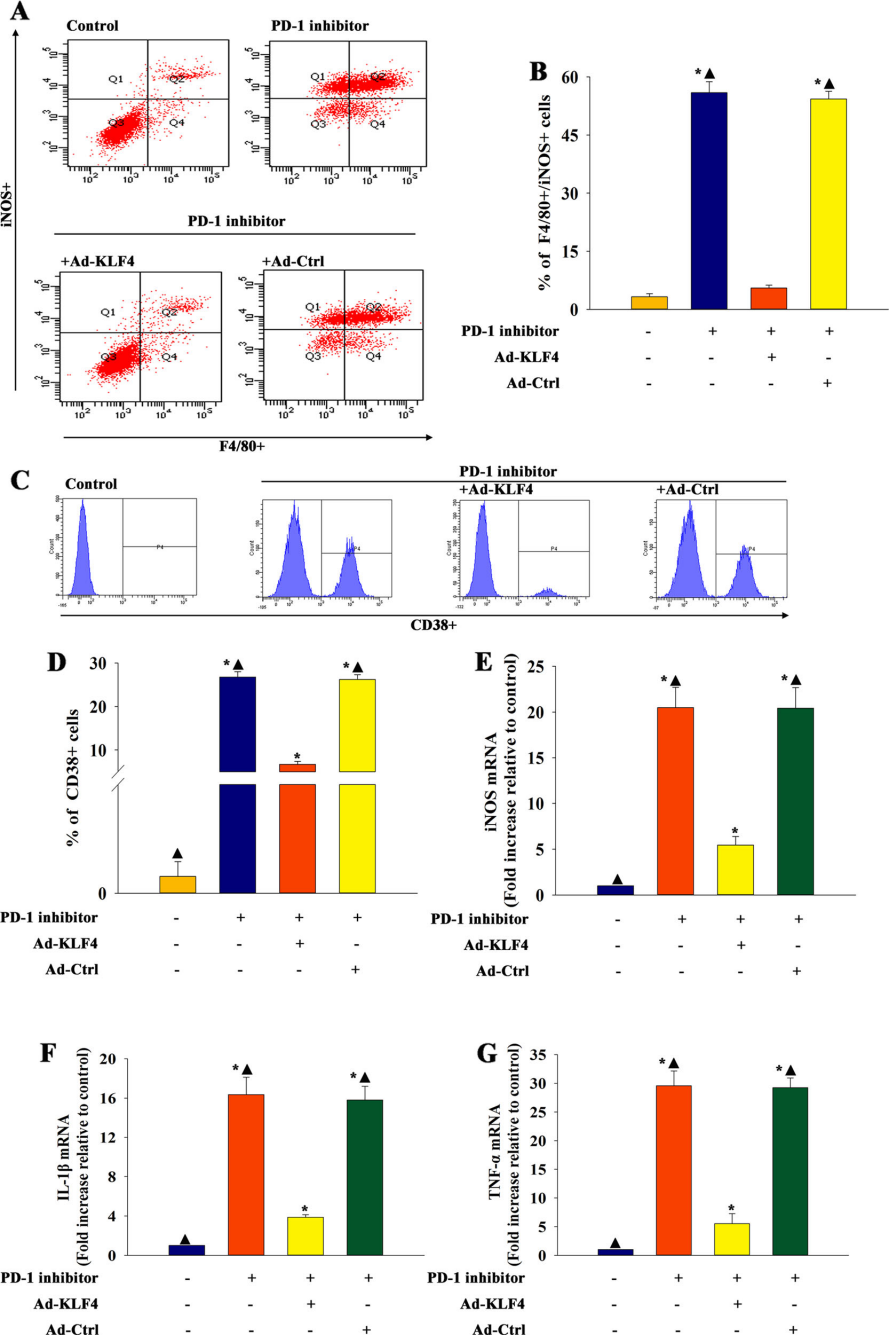
MiR-34a elicited by a PD-1 inhibitor modulated the macrophage phenotype through targeting KLF4. Macrophages transfected with Ad-KLF4 or Ad-Ctrl and treated with a PD-1 inhibitor. Macrophages were treated with a PD-1 inhibitor. Untreated macrophages were used as a control. **a** The typical results of F4/80+/iNOS+ type using flow cytometry in macrophages. **b** Quantitative analysis of F4/80+/iNOS+ cells were analyzed using flow cytometry. **c** The typical results of CD38+ type using flow cytometry. **d** Quantitative analysis of CD38+ cells were analyzed using flow cytometry. *n* = 3 per group. **e**–**g** The expression levels of M1 markers iNOS **e**, IL-1β **f**, and TNF-ɑ **g** mRNAs were examined using qRT-PCR. *n* = 6 per group. ^*^P < 0.05 versus the control group; ^▲^*P* < 0.05 versus the PD-1 inhibitor + Ad-KLF4 group.

The study investigated the target genes of miR-34a regulation using a bioinformatics database to better understand how miR-34a modulated macrophage phenotypes. A putative binding site was identified between miR-34a and KLF4 (Fig. 6a), which was confirmed by dual-luciferase gene reporter assay. The relative luciferase activity was significantly weakened in the KLF4-WT + miR-34a mimic group (Fig. 6b). The protein levels of KLF4 were significantly reduced in macrophages treated with a PD-1 inhibitor, but recovered by an miR-34a inhibitor; however, no difference was detected in macrophages treated with an miR-NC inhibitor (Fig. 6c, d). Subsequently, the study investigated the effects of KLF4 on a PD-1 inhibitor-modulated macrophage phenotype. The in vitro study showed Ad-KLF4 transfection enforced KL4 overexpression in macrophages (Fig. 6e–g). The results suggested that the PD-1 inhibitor increased M1 polarization, whereas the overexpression of KLF4 diminished polarization, which was confirmed by flow cytometry (Fig. 7a–d) and validated by qRT-PCR (Fig. 7e–g). These data suggested that miR-34a was involved in PD-1 inhibitor-mediated macrophage polarization by targeting KLF4-signaling cascades.

## Discussion

ICIs direct the immune system to recognize and target cancer cells. As a novel category of drugs, to some extent, they showed therapeutic effects against cancer^36^. ICIs include programmed cell death-protein 1 inhibitors (anti-PD-1 antibodies: nivolumab, and pembrolizumab); programmed cell death-ligand 1 inhibitors (anti-PD-L1 antibodies: atezolizumab, avelumab, and durvalumab); and cytotoxic T-lymphocyte-associated antigen 4 inhibitors (anti-CTLA-4 antibodies: ipilimumab and tremelimumab)^37^. At present, many patients may be eligible for ICIs therapy^38^, and the use of ICIs is predicted to increase significantly in the coming years^39^. Recently, cardiotoxicity, including myocarditis, heart failure, and cardiac fibrosis, was identified as a side effect of ICIs^40^, but the mechanism of ICIs-related cardiotoxicity remains unclear^41,42^. The key observation in this study was that the PD-1 inhibitor impaired the cardiac function; both systolic and diastolic functions were impaired to some extent.

Myocardial cell injuries are a hallmark of chemotherapy or targeted therapy-induced decompensated heart failure^43,44^. However, cellular proliferation or vitality of cardiomyocytes was not hampered on exposure to a PD-1 inhibitor in vitro. No cardiomyocyte injury has brought the mechanism of such an inflammatory response inducement into question. Consequence of systemic inflammation may account for the inflammatory response. For previous study found that immune-related cardiac injury activated the recruitment of large numbers of inflammatory monocytes, leading to M1 macrophages responsible for the removal of cell debris and necrotic cell clearance^45^. Also, ICIs were found to induce macrophage infiltration in the myocardium, thus leading to myocarditis^7^. Importantly, previous studies found that inappropriate adjustment of macrophage phenotypes and extended inflammation could impair proper tissue remodeling after cardiac injury^17,46^. At present, the modulation of macrophage polarization is a potential therapeutic target for the treatment of cardiac injury^47^. Our data showed that PD-1 inhibitor treatment induced cardiac function injury accompanied by apparent macrophage polarization to a proinflammation M1 phenotype in vivo and in vitro, in accordance with a previous study suggesting that immune-related cardiotoxicity was involved in macrophage polarization^48^. Proinflammatory macrophage expansion (both locally sourced and infiltrating cells) promotes long-term pathological remodeling^49^. As iNOS represents an integral component of the M1 macrophage proinflammatory gene profile^50^, the in vivo results also showed that a PD-1 inhibitor apparently provoked iNOS+ macrophages. M1 macrophages are a significant source of inflammatory cytokines^51^. Thus, an increase in the level of inflammatory cytokines was observed in the setting of a therapeutic PD-1 inhibitor in vivo, indicating a shift in the inflammatory cell composition to one that promoted cardiac inflammation, leading to cardiac injury.

MiRNA biosynthesis is known to be involved in monocyte differentiation and efficient phagocytosis^52^. In the present study, microarray profiling analyses identified differentially expressed miRs in PD-1 inhibitor treated and normal mice hearts, most of which were cardiac pathology related miRs. Among them, cardiac hypotrophy-related miR, microRNA-145^53^, heart failure associated miR, microRNA-221/222 cluster^54^, and cardiac apoptotic relevant miR, microRNA-125b^55^, were upregulated. At meanwhile, miR-34a was chosen, for the reason that it is the miR most apparently upregulated in heart tissues treated with a PD-1 inhibitor, also acted as an important adverse effector in the heart tissue^56^. In vivo and in vitro experiments showed that increased miR-34a expression was closely associated with M1 polarization and metabolic inflammation. A positive correlation between miR-34a expression and macrophage polarization has also been reported in a previous study^57^. The modulation of miR-34a-induced macrophage polarization had an effect on cardiometabolic diseases^58^. In the case of PD-1 inhibitor treatment, the expression of miR-34a elevated in a time-dependent manner, coincident with a previous finding that dead cells were removed by M1 macrophages in a time-dependent manner^59^. The precise mechanism by which a PD-1 inhibitor induced the upregulation of miR-34a expression remains unknown. However, the in vivo results suggested that the inhibition of miR-34a not only recovered the cardiac function impaired by a PD-1 inhibitor but also effectively suppressed proinflammatory polarization of macrophages. Moreover, the in vitro inhibition of miR-34a effectively suppressed M1 polarization. The present study indicated that miR-34a inhibition might have therapeutic potential by modulating macrophage phenotypes, thus resolving inflammation and restoring cardiac function impaired by a PD-1 inhibitor.

Macrophages exhibit remarkable plasticity that allows them to reversibly adapt themselves to changing environmental signals^60^. KLF4 not only serves as a cellular stress sensor but also has an important role in the suppression of inflammatory responses^61^. KLF4 can attenuate inflammatory responses by modulating macrophage polarization^62^, as the activation of KLF4 exerts some potential protective functions in an inflammation-related cardiac injury by modulating macrophage polarization^25^. The present study found that the PD-1 inhibitor decreased the expression level of KLF4, accompanied by the promotion of M1 polarization, enforced increase in the expression level of KLF4 in vitro, diminished the pro-M1 polarization effect of the PD-1 inhibitor. This study further found that the inhibition of miR-34a in macrophages recovered the impaired expression of KLF4 induced by the PD-1 inhibitor in vitro. The effects were similar to those in a previous study showing that KLF4, downstream of miR-34a, took part in macrophage polarization to decrease the proinflammation effect^63^.

## Conclusions

This study provided compelling evidence for the role of a PD-1 inhibitor in polarizing macrophages to a proinflammation phenotype that accounted for cardiac function injury. Specifically, it demonstrated that miR-34a was a key factor conferring cardiac injury by targeting KLF4 to induce a proinflammation polarization state. These data helped understand the pathogenesis of cardiac injury during immunotherapy. The findings might provide new targets in ameliorating cardiac injury in patients who received immunotherapy.

## Supplementary information

Supplementary Data

Supplementary Figure2

Supplementary Figure3

Supplementary Figure legends4
