# Supplementary material for: Immune checkpoint inhibitor induces cardiac injury through polarizing macrophages via modulating microRNA-34a/Kruppel-like factor 4 signaling
Source: Cell Death Dis. 2020 Jul 24;11(7):575. doi: 10.1038/s41419-020-02778-2 (PMC7382486; doi:10.1038/s41419-020-02778-2)
Supplement: Supplementary file 1 — Supplementary Data [file 41419_2020_2778_MOESM1_ESM.doc]

**Supplemental data 1. Values of iNOS expression in each animal per group (Fig.1).**

| **iNOS** |
| --- |
| **Control 1 0.1346** |
| **Control 2 0.1533** |
| **Control 3 0.1002** |
| **PD-1 inhibitor 1 0.3940** |
| **PD-1 inhibitor 2 0.4112** |
| **PD-1 inhibitor 3 0.3722** |

**Supplemental data 2. Values of iNOS expression in each animal per group (Fig.3).**

| **iNOS** |
| --- |
| **Control 1 0.06423** |
| **Control 2 0 .1023** |
| **Control 3 0.0822** |
| **PD-1 inhibitor 1 0.3884** |
| **PD-1 inhibitor 2 0.4109** |
| **PD-1 inhibitor 3 0.3711** |
| **PD-1 inhibitor**  **+miR-34a inhibitor 1 0.1778** |
| **PD-1 inhibitor**  **+miR-34a inhibitor 2 0.2011** |
| **PD-1 inhibitor**  **+miR-34a inhibitor 3 0.1622** |
| **PD-1 inhibitor**  **+miR-NC inhibitor 1 0.3811** |
| **PD-1 inhibitor**  **+miR-NC inhibitor 2 0.4022** |
| **PD-1 inhibitor**  **+miR-NC inhibitor 3 0.3622** |

**Supplemental data 3. Values of each markers in each group.**

| **F4/80+/iNOS+ CD38+ CD206+** |
| --- |
| **Control 1 3.30 0.10 0.40** |
| **Control 2 4.00 0.20 0.50** |
| **Control 3 2.40 0.05 0.60** |
| **PD-1 inhibitor 1 50.20 25.40 0.60** |
| **PD-1 inhibitor 2 55.34 26.50 0.40** |
| **PD-1 inhibitor 3 52.09 27.10 0.70** |
|  |
| **F4/80+/iNOS+ CD38+** |
| **Control 1**  **2.60 0.10** |
| **Control 2 2.00 0.30** |
| **Control 3 3.40 0.05** |
| **PD-1 inhibitor 1 52.50 26.40** |
| **PD-1 inhibitor 2 50.33 27.90** |
| **PD-1 inhibitor 3 55.09 25.10** |
| **PD-1 inhibitor+**  **miR-34a inhibitor 1 3.46 6.20** |
| **PD-1 inhibitor+**  **miR-34a inhibitor 2 4.09 6.80** |
| **PD-1 inhibitor+**  **miR-34a inhibitor 3 5.33 7.00** |
| **PD-1 inhibitor+**  **miR-NC inhibitor 1 55.45 26.00** |
| **PD-1 inhibitor+**  **miR-NC inhibitor 2 51.23 25.10** |
| **PD-1 inhibitor+**  **miR-NC inhibitor 3 49.35 28.10** |
|  |
| **F4/80+/iNOS+ CD38+** |
| **Control 1**  **4.10 0.10** |
| **Control 2 3.00 0.05** |
| **Control 3 2.60 0.31** |
| **PD-1 inhibitor 1 59.00 26.60** |
| **PD-1 inhibitor 2 55.34 28.10** |
| **PD-1 inhibitor 3 53.44 25.70** |
| **PD-1 inhibitor+**  **Ad-KLF4 1 4.70 6.40** |
| **PD-1 inhibitor+**  **Ad-KLF4 2 5.80 6.20** |
| **PD-1 inhibitor+**  **Ad-KLF4 3 6.00 7.50** |
| **PD-1 inhibitor+**  **Ad-Ctrl 1 54.33**   **25.20** |
| **PD-1 inhibitor+**  **Ad-Ctrl 2 52.34 27.40** |
| **PD-1 inhibitor+**  **Ad-Ctrl 3 56.30 26.10** |
