# Supplementary figures and images for: Immune checkpoint inhibitor induces cardiac injury through polarizing macrophages via modulating microRNA-34a/Kruppel-like factor 4 signaling
Source: Cell Death Dis. 2020 Jul 24;11(7):575. doi: 10.1038/s41419-020-02778-2 (PMC7382486; doi:10.1038/s41419-020-02778-2)

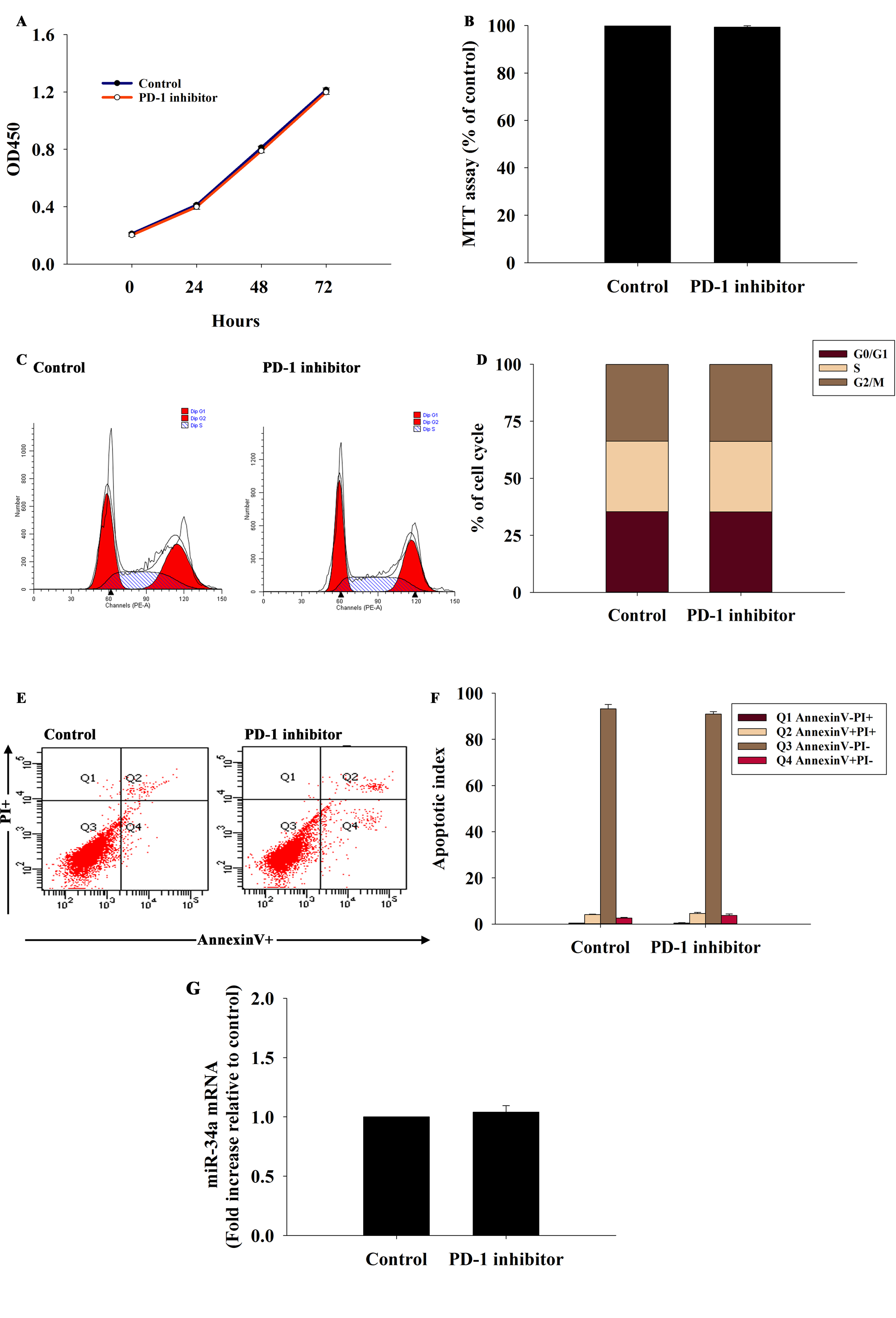

Supplement: Supplementary file 2 — Supplementary Figure2 [file 41419_2020_2778_MOESM2_ESM.tif]

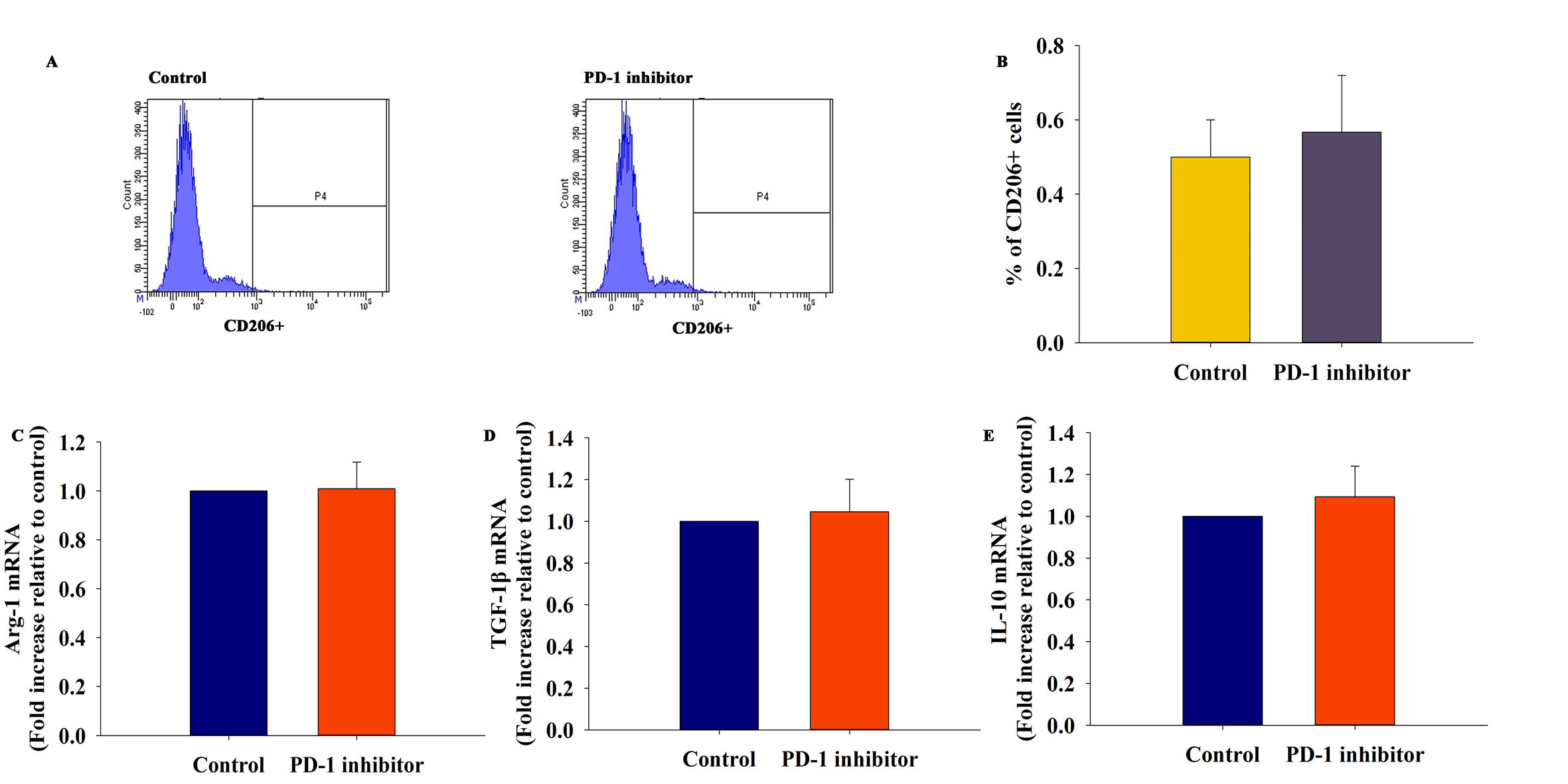

Supplement: Supplementary file 3 — Supplementary Figure3 [file 41419_2020_2778_MOESM3_ESM.tif]
