# Supplementary material for: Immune checkpoint inhibitor induces cardiac injury through polarizing macrophages via modulating microRNA-34a/Kruppel-like factor 4 signaling
Source: Cell Death Dis. 2020 Jul 24;11(7):575. doi: 10.1038/s41419-020-02778-2 (PMC7382486; doi:10.1038/s41419-020-02778-2)
Supplement: Supplementary file 4 — Supplementary Figure legends4 [file 41419_2020_2778_MOESM4_ESM.docx]

**Figure. S.1. PD-1 inhibitor does not cause cardiomyocyte injury.** (A) Proliferation growth curves of HL-1 cells incubated with a PD-1 inhibitor were determined using the CCK-8 proliferation assay. n=3 per group. (B) A PD-1 inhibitor was added to the culture media, and cell viability was analyzed using the MTT assay. n=3 per group. (C and D) Cell cycle distribution was analyzed. n=3 per group. (E and F) Representative flow cytometric dot plots of apoptotic cells after Annexin V/propidium iodide staining. n=3 per group. (G) Expression of miR-34 in the HL-1 cells incubated with a PD-1 inhibitor was determined using qRT-PCR. n=3 per group. Data is representative of mean ± standard deviation from three independent experiments.

**Figure. S.2. PD-1 inhibitor does not polarize the macrophages to M2 phenotype.** (A) The typical results of CD206+ type using flow cytometry in macrophages treated with conditioned medium with or without a PD-1 inhibitor. (B) Quantitative analysis of results in M2 macrophages analyzed using flow cytometry. n=3 per group. The expression levels of M2 markers Arg1 (C), TGF-β1 (D), and IL-10 (E) mRNAs were examined using qRT-PCR. n=6 per group.
